# A Miniaturized QEPAS Trace Gas Sensor with a 3D-Printed Acoustic Detection Module

**DOI:** 10.3390/s17081750

**Published:** 2017-07-31

**Authors:** Xiaotao Yang, Youhong Xiao, Yufei Ma, Ying He, Frank K. Tittel

**Affiliations:** 1 College of Power and Energy Engineering, Harbin Engineering University, Harbin 150001, China; yangxiaotao@hrbeu.edu.cn; 2 National Key Laboratory of Science and Technology on Tunable Laser, Harbin Institute of Technology, Harbin 150001, China; hearkenyi@hit.edu.cn; 3 Department of Electrical and Computer Engineering, Rice University, 6100 Main Street, Houston, TX 77005, USA; fkt@rice.edu

**Keywords:** QEPAS, 3D printing, miniaturization, C_2_H_2_ quantification

## Abstract

A 3D printing technique was introduced to a quartz-enhanced photoacoustic spectroscopy (QEPAS) sensor and is reported for the first time. The acoustic detection module (ADM) was designed and fabricated using the 3D printing technique and the ADM volume was compressed significantly. Furthermore, a small grin lens was used for laser focusing and facilitated the beam adjustment in the 3D-printed ADM. A quartz tuning fork (QTF) with a low resonance frequency of 30.72 kHz was used as the acoustic wave transducer and acetylene (C_2_H_2_) was chosen as the analyte. The reported miniaturized QEPAS trace gas sensor is useful in actual sensor applications.

## 1. Introduction

Due to the advantages of highly sensitive, non-invasive, in situ, real-time observations, laser gas sensing methods have attracted a wide range of interest in recent years and are extensively used for environment monitoring, combustion diagnosis, biomedical science, and industrial process control [1,2,3]. Among these methods, photoacoustic spectroscopy (PAS) is one of the most attractive gas sensor techniques, and it is based on the photoacoustic (PA) effect. In the PAS method, a laser source is used to periodically heat a gas sample when the laser emission wavelength matches an absorption line of the gas species. The pressure wave resulting from the non-radiative processes yields an indirect measure of the relative gas concentration. The resulting pressure modulation is detected as acoustic waves using a microphone. However, the acoustic emission signal in PAS is exceedingly weak. Thus, a resonator with a higher resonant Q factor to amplify the signal is essential. However, the size of a typical photoacoustic resonator is relatively large [4].

The quartz-enhanced photoacoustic spectroscopy (QEPAS) technique is an improvement of the PAS method [5]. Unlike a microphone, in QEPAS a piezoelectric quartz tuning fork (QTF) is used as the acoustic wave transducer [6]. The QTF has extremely low internal losses and the Q factor is ~10,000 at 1 atm. Furthermore, it has an acoustic quadrupole geometry. This means it has low sensitivity to external sound [7]. Due to the merit of high sensitivity, the QEPAS technique has been used for the detection of numerous gases, such as carbon disulfide (CS_2_), nitrous acid (HONO), hydrogen sulfide (H_2_S), ethylene (C_2_H_4_) and hydrogen chloride (HCl) [8,9,10,11,12].

In contrast to traditional microphone-based PAS, in QEPAS a size limitation of the gas cell no longer exists. Therefore, a QEPAS sensor gas cell can be designed as described in [13,14,15,16]. In this paper, a novel 3D printing technique was introduced to the QEPAS sensor platform for the first time. The acoustic detection module (ADM) was designed and fabricated using a 3D printing technique and the volume of the ADM was reduced significantly. A small grin lens was used for laser focusing and facilitated laser beam adjustment in the 3D-printed ADM. Such a miniaturized QEPAS trace gas sensor is beneficial in future trace gas-sensing applications.

## 2. Experimental Setup

**Figure 1:**
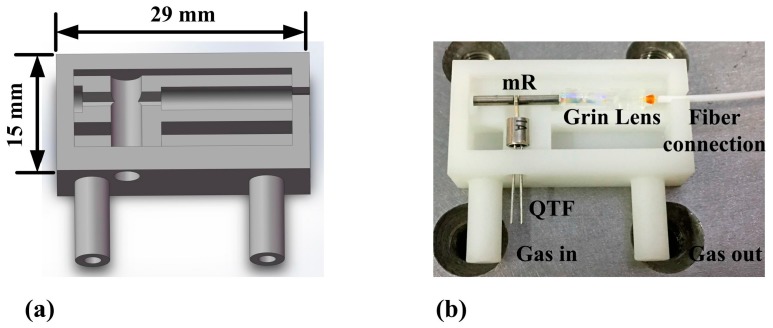
(**a**) 3D model; (**b**) 3D-printed ADM containing QTF, grin lens and mR.

The 3D printing technique offers ease of fabrication. Therefore, several parts of the ADM can be fabricated as an entire device. UV-curable resin was used as the material in the 3D printing fabrication process. The ADM, together with the 3D model from which it was printed, is depicted in Figure 1. For the ADM a smaller volume is desirable. A key attribute in the design is the ability to correctly align the collimated laser beam along the longitudinal axis. The 3D-printed ADM section has a length of 29 mm and a width of 15 mm. A small grin lens with a diameter of 1.8 mm made from borosilicate glass was used for laser focusing. The laser beam was focused between the QTF prongs and passed through the micro-resonator (mR) without touching. The total weight of the 3D-printed ADM containing the QTF, grin lens and mR was only 3 g.

**Figure 2:**
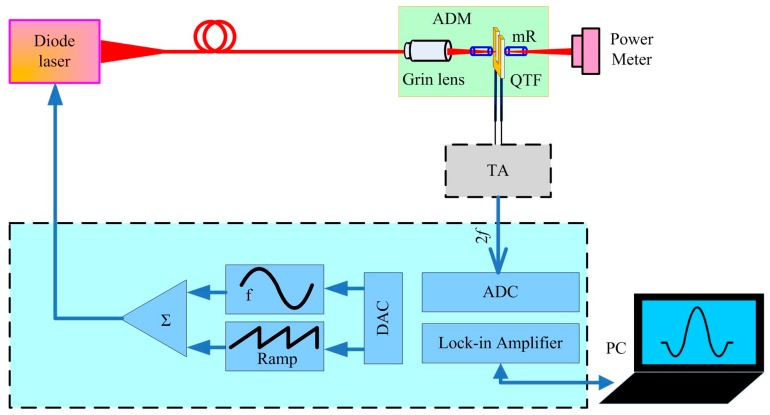
Schematic of the developed QEPAS sensor setup.

A QTF with a low resonant frequency *f*_0_ is beneficial to increase the QEPAS sensor signal [17]. Therefore, in this paper, different from normally used QTFs with *f*_0_ values of ~32.76 kHz, a QTF with an *f*_0_ value of 30.72 kHz was used as an acoustic wave transducer. Acetylene (C_2_H_2_) was chosen as the analyte due to its important applications in the detection of fault gases in transformers and in ethylene streams for polyethylene production [18,19]. The schematic of the developed QEPAS sensor setup is shown in Figure 2. Since the PA effect induces a secondary signal, it is a zero-baseline technique. This means that no signal is produced in the absence of an absorbing species. A low-noise trans-impedance amplifier (TA) with a 10 MΩ feedback resistor converted the current signal produced from the QTF into a voltage. The lock-in amplifier had an integration time of 1 s.

**Figure 3:**
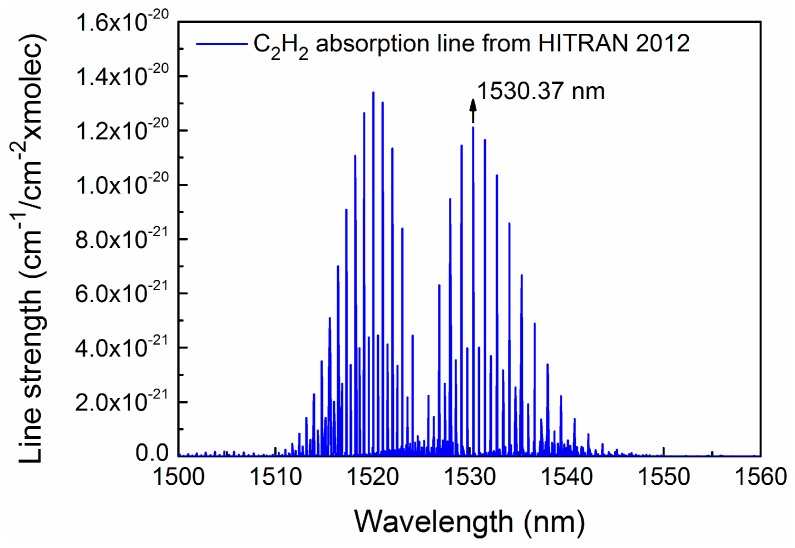
Simulation of C_2_H_2_ absorption lines at the 1.53 μm spectral region based on HITRAN 2012 database.

**Figure 4:**
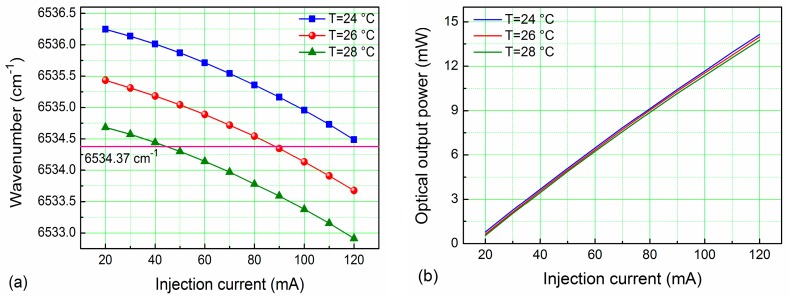
The 1.53 μm CW DFB diode laser output performance: (**a**) wavelength at different TEC temperatures and injection currents; (**b**) optical output power at different TEC temperatures and injection currents.

A pigtailed, near-infrared, continuous-wave (CW), distributed feedback (DFB) diode laser emitting at 1.53 μm was employed as the excitation source. The DFB diode laser was mounted in a 14-pin butterfly package that included a thermoelectric controller (TEC). A 1530.37 nm (6534.37 cm^−1^) absorption line was selected as one of the strongest in the 1.53 μm spectral range for C_2_H_2_ detection using the HITRAN 2012 database [20], which is shown in Figure 3. This absorption line is free from spectral interference of other molecules. The output wavelength of the diode laser targeted the 1530.37 nm absorption line by controlling the temperature of the TEC and the injection current. This was accomplished by setting the diode laser temperature and current to 26 °C and 93 mA, respectively. The experimentally determined temperature and current tuning coefficients were −0.40 cm^−1^/°C and −0.017 cm^−1^/mA, respectively. The performance of this DFB CW diode laser is shown in Figure 4.

## 3. Results and Discussion

**Figure 5:**
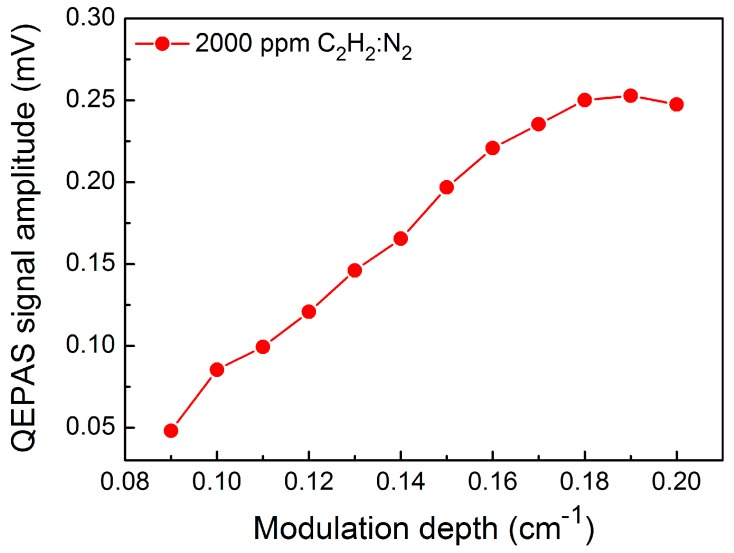
C_2_H_2_-QEPAS signal amplitude as a function of the modulation depth.

The C_2_H_2_-QEPAS sensor performance was evaluated using 2000 ppmv (parts in 10^6^ by volume) C_2_H_2_ in nitrogen (N_2_). The flow rate was controlled to be 120 cm^3^/min using a mass flowmeter. The measurements were performed under laboratory conditions (i.e., atmospheric pressure and room temperature). An optimum distance between the top of QTF prongs and the diode laser beam of 0.7 mm was chosen to achieve the maximum QEPAS signal amplitude [21]. Furthermore, the laser wavelength modulation depth was optimized. The relationship between the QEPAS signal amplitude and the laser wavelength modulation depth for a 2000 ppm C_2_H_2_:N_2_ mixture is shown in Figure 5. Initially, the QEPAS signal increased when the modulation depth increased, and did not change appreciably when the modulation depth was >0.18 cm^−1^. 

**Figure 6:**
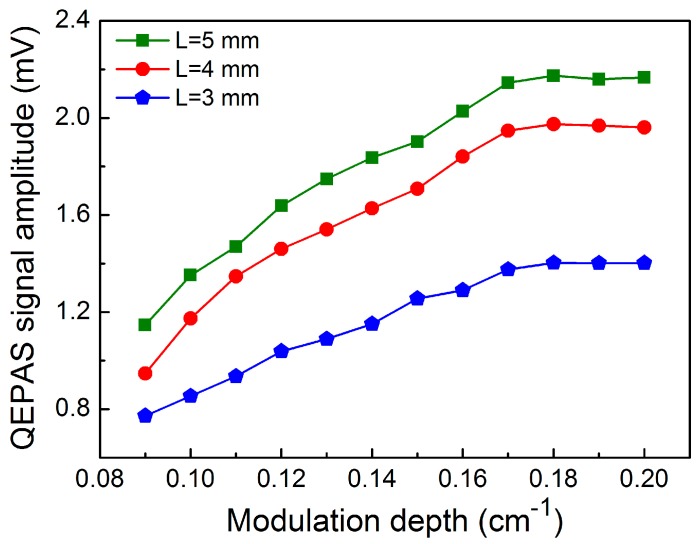
C_2_H_2_-QEPAS signal amplitude as a function of modulation depth for three mRs with different *L* values.

**Figure 7:**
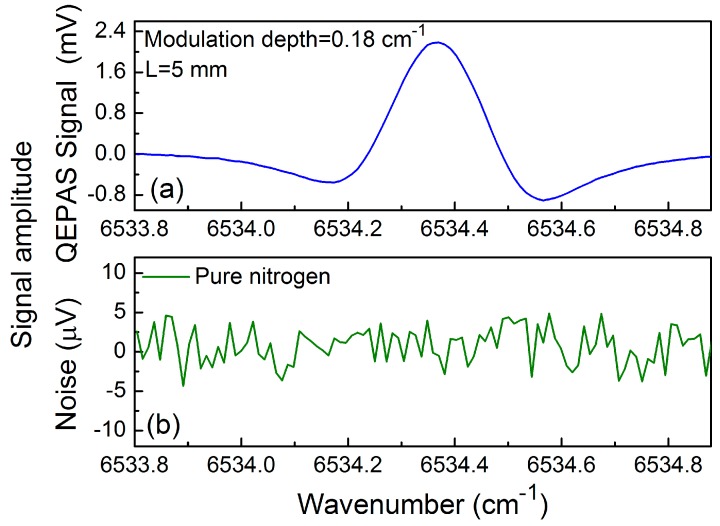
Signal amplitude: (**a**) 2*f* C_2_H_2_-QEPAS signal using mR with *L* = 5 mm at a modulation depth of 0.18 cm^−1^; (**b**) pure N_2_ for noise determination.

When two metallic tubes are added to both sides of the QTF in order to provide a micro-resonator (mR), the QEPAS sensor signal is improved significantly. The optimum length *L* of the mR tubes should be in the range of *λ_s_*/4 < *L* < *λ_s_*/2, where *λ_s_* is the sound wavelength. For a sound velocity of 340 m/s and a frequency of sound of 30.72 kHz, the optimum length of the mR tubes should be in the range of 2.8 mm < *L* < 5.5 mm. In the following experiments, the different lengths of 3, 4 and 5 mm were investigated, respectively, and the inner diameter of the stainless tubes was 0.5 mm. The measured 2*f* C_2_H_2_-QEPAS signal amplitude as a function of the modulation depth when different mRs were employed is shown in Figure 6. The maximum signal enhancement was obtained when *L* = 5 mm and a nine-fold improvement was achieved at this condition. For a modulation depth of 0.18 cm^−1^ and *L* = 5 mm, the measured 2*f* QEPAS signals are shown in Figure 7a. When the ADM was filled with ultra-high-purity nitrogen (N_2_), the background noise of the QEPAS sensor was determined. Figure 7b shows the measured results. The background signal was 2.2 μV. The 1σ minimum detection limit (MDL) of the C_2_H_2_-QEPAS sensor was 2.0 ppm for a 1 s time constant of the lock-in amplifier based on the data depicted in Figure 7. 

## 4. Conclusions

In conclusion, a 3D printing technique was introduced to a QEPAS-based trace gas sensor for the first time. The ADM was designed and fabricated using a 3D printing technique and the ADM volume was compressed significantly. Furthermore, a tiny grin lens was used for laser focusing and facilitated the beam adjustment in the 3D-printed ADM. The total weight of the 3D-printed ADM containing the QTF, grin lens and mR was only 3 g. C_2_H_2_ was chosen as the analyte and a QTF with a low resonance frequency of 30.72 kHz was used as the acoustic wave transducer. After optimization of the modulation depth and the length of the mR tubes, a MDL of 2.0 ppm for the C_2_H_2_-QEPAS sensor was obtained when the modulation depth was 0.18 cm^−1^ and the length of the mR tube was 5 mm. The miniaturized design method reported in this paper will be useful in certain applications of a QEPAS trace gas sensor.
